# Bias and accuracy of dairy sheep evaluations using BLUP and SSGBLUP with metafounders and unknown parent groups

**DOI:** 10.1186/s12711-020-00567-1

**Published:** 2020-08-12

**Authors:** Fernando L. Macedo, Ole F. Christensen, Jean-Michel Astruc, Ignacio Aguilar, Yutaka Masuda, Andrés Legarra

**Affiliations:** 1 GenPhySE, INRAE, 31326 Castanet Tolosan, France; 2 Facultad de Veterinaria, UdelaR, A. Lasplaces 1620, Montevideo, Uruguay; 3 Center for Quantitative Genetics and Genomics, Blichers Allé 20, 8830 Tjele, Denmark; 4 Institut de l’Elevage, CS52627, 31326 Castanet Tolosan, France; 5 Instituto Nacional de Investigación Agropecuaria, Montevideo, Uruguay; 6 Department of Animal and Dairy Science, University of Georgia, Athens, GA USA

## Abstract

**Background:**

Bias has been reported in genetic or genomic evaluations of several species. Common biases are systematic differences between averages of estimated and true breeding values, and their over- or under-dispersion. In addition, comparing accuracies of pedigree versus genomic predictions is a difficult task. This work proposes to analyse biases and accuracies in the genetic evaluation of milk yield in Manech Tête Rousse dairy sheep, over several years, by testing five models and using the estimators of the linear regression method. We tested models with and without genomic information [best linear unbiased prediction (BLUP) and single-step genomic BLUP (SSGBLUP)] and using three strategies to handle missing pedigree [unknown parent groups (UPG), UPG with QP transformation in the ${\mathbf{H}}$ matrix (EUPG) and metafounders (MF)].

**Methods:**

We compared estimated breeding values (EBV) of selected rams at birth with the EBV of the same rams obtained each year from the first daughters with phenotypes up to 2017. We compared within and across models. Finally, we compared EBV at birth of the rams with and without genomic information.

**Results:**

Within models, bias and over-dispersion were small (bias: 0.20 to 0.40 genetic standard deviations; slope of the dispersion: 0.95 to 0.99) except for model SSGBLUP-EUPG that presented an important over-dispersion (0.87). The estimates of accuracies confirm that the addition of genomic information increases the accuracy of EBV in young rams. The smallest bias was observed with BLUP-MF and SSGBLUP-MF. When we estimated dispersion by comparing a model with no markers to models with markers, SSGBLUP-MF showed a value close to 1, indicating that there was no problem in dispersion, whereas SSGBLUP-EUPG and SSGBLUP-UPG showed a significant under-dispersion. Another important observation was the heterogeneous behaviour of the estimates over time, which suggests that a single check could be insufficient to make a good analysis of genetic/genomic evaluations.

**Conclusions:**

The addition of genomic information increases the accuracy of EBV of young rams in Manech Tête Rousse. In this population that has missing pedigrees, the use of UPG and EUPG in SSGBLUP produced bias, whereas MF yielded unbiased estimates, and we recommend its use. We also recommend assessing biases and accuracies using multiple truncation points, since these statistics are subject to random variation across years.

## Background

Genetic progress in selection schemes depends on using correct models for genetic evaluation. Models are simplifications of reality and never completely perfect, which is why tools to analyze systematic errors are necessary. There are three important aspects to check in genetic evaluations: bias, dispersion and accuracy. Bias $\left( {b_{0} = \bar{\hat{u}} - \bar{u}} \right)$ is the difference between estimated breeding values (EBV) $\hat{u}$ and true breeding values (TBV) $u$ and could lead to over- or under-estimation of genetic trend and to poor selection decisions (for example, selecting too many young individuals instead of keeping old ones). In the same way, on the one hand, values of the slope of the regression of TBV on EBV less than 1 imply over-dispersion of the EBV and could lead to an overestimation of the genetic merit of pre-selected candidates. On the other hand, an unbiased estimate of accuracy (the correlation between TBV and EBV) is important to correctly predict the response to selection.

Bias has been found in genetic evaluations of several species. The use of genomic information in dairy cattle selection is widespread and the existence of bias has been extensively studied (e.g. [1–4]). Bias has also been studied in other species, such as pigs [5], dairy goats [6], turkeys [7] and beef cattle [8–10]. In general, biases decrease with more adequate models. However, all these studies rely on the use of pre-corrected data such as deregressed proofs or daughter yield deviations (DYD), which may give wrong estimates of biases if fixed effects are not well estimated [11].

Studies in France and Spain using DYD detected bias in genetic evaluations of dairy sheep breeds. For example, predictions in Lacaune showed bias and over-dispersion of EBV, with more impact for traits under strong selection [12, 13]. Similar results were obtained for milk yield of Pyrenean dairy sheep breeds [14], although genomic evaluations decreased bias compared to pedigree evaluations. Manech Tête Rousse (MTR) is one of the major French Pyrenean dairy sheep breeds. For this breed, the selection scheme switched to genomic selection in 2018 and it is important to verify the bias, dispersion and accuracies, to avoid poor selection decisions. In particular, the bias detected in [14] is not well understood. However, it is difficult to assess such biases with DYD in dairy sheep, since DYD from “first crops” of 20 to 40 daughters are not very accurate.

Legarra and Reverter [11] described the linear regression method (LR method) to detect bias in genetic evaluations. The advantage of this method is the simplicity of the application; it compares EBV of a group of individuals obtained in different evaluations, with less (“partial”) and more (“whole”) information. Comparing the two subsets of EBV, estimators of bias, dispersion and accuracies (relatives or directs) are easily computed. Therefore, it is easy to analyze a genetic evaluation comparing the results of two consecutive evaluations.

To perform genetic evaluation, it should be possible to include genomic information and also to model missing pedigrees if needed. In this work, we tested models using only pedigree information (best linear unbiased prediction (BLUP) model) or including genomic information (in a single-step genomic BLUP (SSGBLUP) model) and applying different strategies to deal with missing pedigree. Missing pedigree may be a problem in most species—in ruminants, parents may be unrecorded, whereas in monogastric species, new lines may be introduced. If we do not consider this missingness, we are assuming the same genetic mean for all missing parents in the pedigree. In dairy sheep, females born from natural mating usually do not have an assigned sire. However, these natural mating rams are offspring of highly selected artificial insemination (AI) rams and thus their breeding value increases over time. In addition, new flocks that entered the breeding scheme until (roughly) 1990 did not have pedigree data. Two strategies can be used to model the missing pedigree: unknown parent groups (UPG) [15, 16] and metafounders (MF) [17]. There is some evidence that the use of MF improves the performance of genetic evaluation [18], but it has not been systematically studied.

The aim of this work was to analyze bias, dispersion, and accuracies in the genetic evaluation of milk yield of MTR using the LR method with several evaluation models and performed over many truncation points of data. A second aim was to compare different strategies (UPG or MF) to manage missing pedigree in BLUP and SSGBLUP contexts. In this manner, we assessed the genetic evaluation of MTR, addressed the best method to consider missing pedigrees in SSGBLUP, and explored the possibilities of the LR method to discriminate models for prediction.

## Methods

### Records and pedigree

Milk production is recorded by the breeding scheme according to the International Committee for Animal Recording rules. The data that we analyzed were collected between 1978 and 2017 and comprise 1,842,295 performance records and 540,999 individuals in the pedigree, with a generation interval of about 4 years. There are missing parentships, either “sire unknown and dam known” (~ 15% of all animals) or “both sire and dam unknown” (~ 15% of all animals). This situation is particularly important in our case, because if we ignore the missing pedigree, the unknown parents of the more recently improved animals will be assigned to the base population at the beginning of the selection program. As a result, these animals will be unfairly penalized and it will not be possible to correctly model the genetic progress. Thus, we defined 13 UPG (or MF; see later). We computed a crude “number of equivalent records” from the first “offspring” of UPG (disregarding later generations). For instance, an individual with $n$ records contributes $n$ to its ancestor UPG if both parents are unknown and $n/2$ if one parent is known. In all cases, the number of equivalent records was larger than 10,000.

### Genomic information

We included genomic information on 3007 AI males (years of birth from 1999 until 2017), all of which have both parents known and are genotyped with the 50 k Illumina chip OvineSNP50. Only autosomal SNPs were considered. Quality control included individual and marker call rate, minor allele frequency (MAF) higher than 0.05, removal of Mendelian conflicts, deviation from Hardy–Weinberg equilibrium (number of heterozygotes deviating more than 15% from the expectation based on allele frequencies), and heritability of gene content (markers with an estimated heritability < 0.98 and significant p-values of the likelihood ratio test, p < 0.01, were discarded) [19]. After quality control, 37,168 effective SNPs were retained.

### Focal individuals

It is possible to apply the LR method to any group of individuals of a population, provided that they represent a homogenous tier (i.e. they are similarly selected, and prediction at the time of selection is based on the same sources of information). In this work, we were interested in evaluating bias, dispersion and accuracy of males at the time of their selection, i.e. at birth before they have progeny with records. The reason we are interested in this group is that most of the genetic gain in dairy sheep is obtained via males. In total, 10 groups of focal individuals were analyzed; each group corresponding to selected rams born from 2005 to 2014. These males were selected based on parent average to be progeny-tested and thus their genetic variation is smaller than that of their contemporaries [20].

### Estimators of the LR method

In brief, the LR method estimates bias, dispersion and accuracies, based on the comparison of two subsets of EBV, estimated with less and more information, for the same group of individuals. In this paper, we will use the symbols $\hat{u}_{p}$ or EBV_p_ to refer to the EVB estimated with less information (or “partial” dataset) and $\hat{u}_{w}$ or EBV_w_ to refer to the EBV estimated with more information (or “whole” dataset). The LR method presents one estimator for the bias ($\hat{\Delta }_{p}$), one estimator for the dispersion ($\hat{b}_{p}$) and four estimators related to the accuracies ($\rho_{wp}$, $acc_{p}^{2}$, $\rho_{wp}^{2}$, $\widehat{{\varvec{ }rel}}_{p}$). The estimators are summarized below; for a deeper overview and properties of the estimators see [11, 21].

#### Bias ($\hat{\Delta }_{p}$)

The estimator of the bias is obtained from the difference between the mean of EBV_p_ and the mean of EBV_w_, $\hat{\Delta }_{p} = \overline{{\hat{u}_{p} }} - \overline{{\hat{u}_{w} }}$. In absence of bias, the expected value of this estimator is 0.

#### Dispersion ($\hat{b}_{p}$)

The estimator of dispersion of EBV is the slope of the regression of EBV_w_ on EBV_p_, $\hat{b}_{p} = \frac{{cov\left( {\hat{u}_{p} ,\hat{u}_{w} } \right)}}{{var\left( {\hat{u}_{p} } \right)}}$. If over- or under-dispersion does not exists, the expected value of the estimator is 1, values of $\hat{b}_{p} < 1$ indicate over-dispersion whereas values of $\hat{b}_{p} > 1$ indicate under-dispersion.

### Estimators related to accuracies

#### Ratio of accuracies ($\hat{\rho }_{w,p}$)

This estimator estimates the inverse of the relative gain in accuracy from EBV_p_ to EBV_w_. It is the correlation between EBV_p_ and EBV_w_, $\hat{\rho }_{w,p} = \frac{{cov\left( {\hat{u}_{p} ,\hat{u}_{w} } \right)}}{{\sqrt {var\left( {\hat{u}_{p} } \right)var\left( {\hat{u}_{w} } \right)} }}$ and the expected value is $\frac{{acc_{p} }}{{acc_{w} }}$. A high value of this estimator means a small increase in accuracy, whereas a low value means a large increase in accuracy, when we add phenotypic information to genetic evaluations. For instance, a value of 0.7 means that the evaluation with the “partial” dataset is quite similar to the evaluation with the “whole” dataset, i.e. more phenotypes do not add much new information. This can be seen also as the relative increase in accuracy brought by phenotypes is $\frac{1}{{\hat{\rho }_{w,p} }} - 1 = \frac{{acc_{w} - acc_{p} }}{{acc_{p} }}$ (Matias Bermann, University of Georgia, personal communication). Thus, it is expected that genomic evaluations have higher $\hat{\rho }_{w,p}$ than pedigree-based evaluations.

#### Ratio of reliabilities ($\hat{\rho }_{p,w}^{2}$)

This estimator is the slope of the regression of EBV_p_ on EBV_w_, $\hat{\rho }_{p,w}^{2} = \frac{{cov\left( {\hat{u}_{p} ,\hat{u}_{w} } \right)}}{{var\left( {\hat{u}_{w} } \right)}}$ and, similar to the ratio of accuracies, it represents the inverse of the gain in reliabilities from EBV_p_ to EBV_w_. The expected value is $\frac{{acc_{p}^{2} }}{{acc_{w}^{2} }}$.

#### Selected reliability of EBV_p_ ($\widehat{acc}_{p}^{2}$)

In a general formulation, $\widehat{acc}_{p}^{2} = \frac{{cov\left( {\hat{u}_{p} ,\hat{u}_{w} } \right)}}{{\sigma_{g*}^{2} }}$, where $\sigma_{g* }^{2}$ is the genetic variance of the group of individuals of interest. We use this more general formulation as in [21] instead of the formulation used in [11], because the latter is adequate only for a group of animals that represent the whole population after selection. In this work, we analyzed EBV of sets of contemporary young rams of the population, in other words highly-selected individuals, which decreases reliability [20, 21]. A difficulty associated to this estimator is the necessity of an estimation of the genetic variance of a group of individuals. We estimated the genetic variance of each group of focal individuals following [22] using the complete dataset. We used Gibbs sampling with the complete dataset with 150,000 iterations and a burn-in of 15,000 iterations. At each 150-th iteration, we took samples of the EBV of all AI males in the 10 focal groups and we computed, for each of these groups, the variance of these samples. This results in samples from the posterior distributions of the 10 genetic variances, one for each group of AI males.

#### Unselected reliability of EBVp ($\widehat{rel}_{p}$)

This estimator estimates the reliability as if there was no selection, $\widehat{rel}_{p} = 1 - \frac{{\sigma_{g*}^{2} }}{{\sigma_{g}^{2} }}\left( {1 - \widehat{acc}_{p}^{2} } \right)$ as in [23], where $\sigma_{g}^{2}$ is the genetic variance of the base population and $\sigma_{g* }^{2}$ is the genetic variance of the group of individuals of interest (see above). A short derivation of $\widehat{rel}_{p}$ follows from [21, 24], $Cov\left( {\hat{u}_{p} ,\hat{u}_{w} } \right) = \sigma_{g*}^{2} - PEV$, so $PEV = \sigma_{g*}^{2} - Cov\left( {\hat{u}_{p} ,\hat{u}_{w} } \right)$. The reliability that is unaffected by selection is $r^{2} = 1 - \frac{PEV}{{\sigma_{g}^{2} }}$ leading to $r^{2} = 1 - \frac{{\sigma_{g*}^{2} }}{{\sigma_{g}^{2} }}\left( {1 - r^{2*} } \right)$ [23], where $r^{2*}$ is the selected reliability. The reliability $\widehat{rel}_{p}$ can be interpreted as if the focal individuals were not selected, or, in other words, as the average theoretical reliability of the focal individuals obtained from the mixed model equations (MME).

### Data analysis

To apply the LR method, we have to obtain EBV from the partial dataset and the whole dataset. In this work, in order to obtain an empirical distribution of the statistics of the LR method, we performed several comparisons between EBV_p_ and EBV_w_, taking EBV_p_ from rams born in year $y_{p}$ (2005 to 2014) and EBV_w_ from years $y_{p} + 2$ until year 2017 (last year of records for this work). The year of the first set of EBV_w_ was $y_{p} + 2$ because the first daughters of the selected rams generally start to produce 2 years after birth. For example, if we take the EBV at birth of rams born in 2005 as EBV_p_, we have EBV_w_ of these rams from years 2007 to 2017, thus we have 11 sets of estimators; and if we take EBV from rams born in year 2014 as EBV_p_, we only have EBV_w_ from year 2016 to 2017, thus only two sets of estimators. In total, we performed 65 comparisons, e.g. 2005 vs 2007, 2005 vs 2008 ... 2005 vs 2017 … 2014 vs 2016 and 2014 vs 2017.

Bias or accuracies are properties of the partial dataset only, and not of the whole dataset. Sampling several “partial” years allows to describe possible variations due to chance, i.e. properties of BLUP only hold on expectation. In addition, by considering multiple “whole” datasets, we tried to evaluate random deviations of the estimates of biases and accuracies. For instance, a ram may stop getting progeny performances after a few years, yet the estimates of contemporary groups may change. The theory of the LR method (actually, BLUP theory) shows that the estimators of the LR method are correct regardless of whether rams are selected (and having more and more offspring) or not.

We considered several models for the evaluations that are presented below. We applied the LR method within models, with both EBV_p_ and EBV_w_ obtained with the same model. We also applied this method across models: EBV_p_ obtained with one model, for example regular BLUP with MF, and EBV_w_ from another model, for example SSGBLUP with UPG. Finally, because the addition of genomic data to the evaluation can be seen as “more information”, it is possible to see EBV obtained at the same time but without and with genomic information as EBV_p_ and EBV_w_, respectively. Thus, we also compared the EBV of the rams at birth estimated with the BLUP and SSGBLUP models. For example, the EBV of rams at birth in 2005 were estimated with BLUP as EBV_p_ and estimated with SSGBLUP as EBV_w_.

Although there is no theoretical support for using the LR method across models [21], our objective was to check the consistency of models with each other, in the sense that a refinement of the model should not introduce unexpected changes in the evaluations. Otherwise, one of the models could possibly be quite wrong. For instance, switching the genetic evaluation of milk yield from lactational measures to test-day models should not introduce big changes. Likewise, selection schemes that start adding genomic information to the genetic evaluations must change models without too large changes in the EBV. Viewed in this way, it is important to check the coherence (lack of strong changes) from one model to the other. We focused on the regression coefficient $\hat{b}_{p}$, with an expected value of 1.

To summarize the 65 comparisons, raw averages of estimators are not correct because some years are more represented that others, e.g. 2005 has 11 comparisons whereas 2014 has two comparisons. Thus, we used the pseudo-model ${\mathbf{es}}_{pw} = {\mathbf{Xy}}_{p} + {\mathbf{Zy}}_{w} + {\varvec{\upvarepsilon}}$, where ${\mathbf{es}}_{pw}$ is a vector of the 65 values of the estimator ($\hat{\Delta }_{p}$, $\hat{b}_{p}$, $\hat{\rho }_{wp}$, $\widehat{acc}_{wp}^{2}$, $\hat{\rho }_{pw}^{2}$, $\widehat{rel}_{p}$) from the comparison of EBV_p_ of the rams born in year $p$ and of EBV_w_ of same rams obtained in year $w$, ${\mathbf{y}}_{p}$ contains values for years $p$ (2005 to 2014) and ${\mathbf{y}}_{w}$ contains values for years $w$ (${\text{y}}_{\text{p}} + 2$ until 2017), and we report an estimable function that yields $\widehat{{{\mathbf{es}}_{pw} }}$ as if the design was balanced: $\widehat{{{\mathbf{es}}_{pw} }} = \frac{1}{np}1^{\prime}{\hat{\mathbf{y}}}_{p} + \frac{1}{nw}1^{\prime}{\hat{\mathbf{y}}}_{w}$ where $np$ and $nw$ are the number of different years for the “partial” dataset (8) and “whole” dataset (11). The pseudo-model was fit by least squares (lm function in R), and the R package Gmodels version 2.18.1 was used to compute the contrasts. The code is given in “Appendix”.

### Models

The genetic evaluations were performed using the regular linear model for genetic evaluation of MTR. This is a univariate model with repeated records for milk yield that accounts for heterogeneity of variances across contemporary groups [25]:

$${\varvec{\Lambda}}{\mathbf{y}} = {\mathbf{y}}_{c} = {\mathbf{Xb}} + {\mathbf{W}}_{u} {\mathbf{u}} + {\mathbf{W}}_{p} {\mathbf{p}} + {\mathbf{e}},$$

where ${\varvec{\Lambda}}$ is a diagonal matrix of scaling factors for heterogeneity of variances, ${\mathbf{y}}$ is a vector of milk yield records, ${\mathbf{y}}_{c}$ is a vector of the observations corrected for heterogeneity of variances, ${\mathbf{b}}$ is a vector of the fixed effects: contemporary group, age and number of lactation, month of lambing and interval “from lambing to first milk recording”, ${\mathbf{u}}$ is a vector of breeding values, ${\mathbf{p}}$ is a vector of permanent animal effects, ${\mathbf{e}}$ is a vector of residuals, and ${\mathbf{X}}$, ${\mathbf{W}}_{p}$ and ${\mathbf{W}}_{u}$ are incidence matrices for fixed effects, permanent animal effects, and breeding values. Following [25], the $i{\text{th}}$ diagonal element in ${\varvec{\Lambda}}$ is $\exp \left( {\frac{{\tau_{i} }}{2}} \right)$; a scaling factor for fixed and random effects. The linear model for $\tau_{i} = {\mathbf{S}}_{i} {\varvec{\upbeta}}$, where ${\varvec{\upbeta}}$ is the vector of unknown effects for year (fixed) and flock-year (random) and ${\mathbf{S}}_{i}$ is the design vector. Heritability was fixed at 0.30 (the value used in official evaluations; an estimate calculated with the complete dataset was equal to 0.28). In models with UPG, EBV cannot be estimated, and the genetic basis changes with the model used. Therefore, we referred all estimates of EBV to the average EBV of the females born in 2005. Using this animal model, different (sub) models were defined depending on: (1) the use or not of genomic information, and (2) the strategy to model missing pedigree.

We used BLUP models with the matrix of additive genetic relationships ${\mathbf{A}}$ [24] and models that include the genomic information in a single step (SSGBLUP). The SSGBLUP models replaces ${\mathbf{A}}$ with a matrix ${\mathbf{H}}$. that combines pedigree and genomic relationships [26–28].

To model the missing pedigree, we used three strategies, unknown parent groups for ${\mathbf{A}}$ (UPG) and for ${\mathbf{H}}$ (EUPG) and metafounders (MF). Unknown parents groups were developed to avoid bias due to differences in genetic means of groups of individuals with different origins [15, 29]. The theory of UPG adapted to SSGBLUP models was reviewed by [16]. Later, Legarra et al. [17] conceived the theory of MF that represents base populations by related, inbred pseudo-individuals. The aim of MF was to provide a coherent theory, where UPG would account for the reduction in genetic variance due to drift and for relationships across base populations. Using genomic information, it is possible to estimate the relatedness between groups of unknown parents (${\varvec{\Gamma}}$ matrix) [17, 30], and this relationship matrix across MF can be used also in purely pedigree-based BLUP models. We estimated matrix ${\varvec{\Gamma}}$ from observed genotypes using the GLS method of [30].

Let index 0 denote the base populations (either UPG or MF), index 1 “non-genotyped animals”, and index 2 “genotyped animals”. Denote $${\mathbf{A}}^{ - 1} = \left( {\begin{array}{*{20}c} {{\mathbf{A}}^{11} } & {{\mathbf{A}}^{12} } \\ {{\mathbf{A}}^{21} } & {{\mathbf{A}}^{22} } \\ \end{array} } \right)$$ as the usual inverse of the relationship matrix and ${\mathbf{A}}_{22}^{ - 1}$ the inverse including only genotyped animals, $${\mathbf{A}}^{*} = \left( {\begin{array}{*{20}c} {{\mathbf{A}}^{00} } & {{\mathbf{A}}^{01} } & {{\mathbf{A}}^{02} } \\ {{\mathbf{A}}^{10} } & {{\mathbf{A}}^{11} } & {{\mathbf{A}}^{12} } \\ {{\mathbf{A}}^{20} } & {{\mathbf{A}}^{21} } & {{\mathbf{A}}^{22} } \\ \end{array} } \right)$$ as the generalized inverse (as it is not full rank) including UPG, and $${\mathbf{A}}^{\left( \varGamma \right) - 1} = \left( {\begin{array}{*{20}c} {{\mathbf{A}}^{\left( \varGamma \right)00} } & {{\mathbf{A}}^{\left( \varGamma \right)01} } & {{\mathbf{A}}^{\left( \varGamma \right)02} } \\ {{\mathbf{A}}^{\left( \varGamma \right)10} } & {{\mathbf{A}}^{\left( \varGamma \right)11} } & {{\mathbf{A}}^{\left( \varGamma \right)12} } \\ {{\mathbf{A}}^{\left( \varGamma \right)20} } & {{\mathbf{A}}^{\left( \varGamma \right)21} } & {{\mathbf{A}}^{\left( \varGamma \right)22} } \\ \end{array} } \right)$$ as the inverse using MF. All three matrices are easily built using simple modifications of Henderson’s algorithm [31].

The SSGBLUP model proceeds by modifying the conditional variances and covariances in the inverse matrices according to observed genomic information, by obtaining ${\mathbf{H}}^{ - 1}$ matrices from ${\mathbf{A}}^{ - 1}$ matrices. Corresponding matrices are, for SSGBLUP-UPG:

$${\mathbf{H}}_{{{\mathbf{UPG}}}}^{*} = \left( {\begin{array}{*{20}c} {{\mathbf{A}}^{00} } & {{\mathbf{A}}^{01} } & {{\mathbf{A}}^{02} } \\ {{\mathbf{A}}^{10} } & {{\mathbf{A}}^{11} } & {{\mathbf{A}}^{12} } \\ {{\mathbf{A}}^{20} } & {{\mathbf{A}}^{21} } & {{\mathbf{A}}^{22} } \\ \end{array} } \right) + \left( {\begin{array}{*{20}c} 0 & 0 & 0 \\ 0 & 0 & 0 \\ 0 & 0 & {{\mathbf{G}}^{ - 1} - {\mathbf{A}}_{22}^{ - 1} } \\ \end{array} } \right),$$

where ${\mathbf{G}}$ is the genomic relationship matrix that is built following the first method in [32], using observed allele frequencies, and made comparable to ${\mathbf{A}}_{22}$ following [5].

It is well known that this matrix is, at best, an approximation [16] because the theory of matrix ${\mathbf{H}}$ was derived under the constraint that ${\mathbf{A}}$ is full rank, which is not the case for ${\mathbf{A}}^{*}$. The same authors in [16] proposed a full transformation hereafter called “exact UPG” (EUPG) that can be written as:

$${\mathbf{H}}_{EUPG}^{*} = \left( {\begin{array}{*{20}c} {{\mathbf{A}}^{00} } & {{\mathbf{A}}^{01} } & {{\mathbf{A}}^{02} } \\ {{\mathbf{A}}^{10} } & {{\mathbf{A}}^{11} } & {{\mathbf{A}}^{12} } \\ {{\mathbf{A}}^{20} } & {{\mathbf{A}}^{21} } & {{\mathbf{A}}^{22} } \\ \end{array} } \right) + \left( {\begin{array}{*{20}c} {{\mathbf{Q}}_{2}^{'} \left( {{\mathbf{G}}^{ - 1} - {\mathbf{A}}_{22}^{ - 1} } \right){\mathbf{Q}}_{2} } & 0 & { - {\mathbf{Q}}_{2}^{'} \left( {{\mathbf{G}}^{ - 1} - {\mathbf{A}}_{22}^{ - 1} } \right)} \\ 0 & 0 & 0 \\ { - \left( {{\mathbf{G}}^{ - 1} - {\mathbf{A}}_{22}^{ - 1} } \right){\mathbf{Q}}_{2} } & 0 & {{\mathbf{G}}^{ - 1} - {\mathbf{A}}_{22}^{ - 1} } \\ \end{array} } \right),$$

where ${\mathbf{Q}}_{2}$ is the matrix containing UPG compositions for genotyped animals.

Whereas in “regular” SSGBLUP the only changes concern genotyped animals, here there are extensive changes that make programming difficult. In addition, because ${\mathbf{G}}$ accounts correctly for the different origins and does not need pedigree completion, there is, depending on the pedigree structure, some sort of double-counting as observed by [18]. These problems are solved by MF which proposes:

$${\mathbf{H}}_{MF}^{ *} = \left( {\begin{array}{*{20}c} {{\mathbf{A}}^{\left( \varGamma \right)00} } & {{\mathbf{A}}^{\left( \varGamma \right)01} } & {{\mathbf{A}}^{\left( \varGamma \right)02} } \\ {{\mathbf{A}}^{\left( \varGamma \right)10} } & {{\mathbf{A}}^{\left( \varGamma \right)11} } & {{\mathbf{A}}^{\left( \varGamma \right)12} } \\ {{\mathbf{A}}^{\left( \varGamma \right)20} } & {{\mathbf{A}}^{\left( \varGamma \right)21} } & {{\mathbf{A}}^{\left( \varGamma \right)22} } \\ \end{array} } \right) + \left( {\begin{array}{*{20}c} 0 & 0 & 0 \\ 0 & 0 & 0 \\ 0 & 0 & {{\mathbf{G}}_{05}^{ - 1} - {\mathbf{A}}_{22}^{\left( \varGamma \right) - 1} } \\ \end{array} } \right),$$

where ${\mathbf{G}}_{05}$ is built with allele frequencies of 0.5 and there is no extra scaling to match ${\mathbf{A}}_{22}^{\left( \varGamma \right)}$, although there is blending as described below.

For all SSGBLUP models, the blending between ${\mathbf{G}}$ and ${\mathbf{A}}_{22}$ or between ${\mathbf{G}}_{05}$ and ${\mathbf{A}}_{22}^{\left( \varGamma \right)}$ was done using 0.95 and 0.05, as respective weights [32–34]. An analysis using MF also needs to consider that the population is more related by construction. We used the scaling of genetic variance in [17] such that if the genetic variance considering BLUP_UPG was $\sigma_{u}^{2}$, the genetic variance component attributed to ${\mathbf{H}}_{MF}^{*}$ was $\sigma_{u}^{2} /k$ for $k = 1 + \frac{{\overline{{diag\left( {\varvec{\Gamma}} \right)}} }}{2} - {\bar{\mathbf{\varGamma }}}$.

Now we can describe the five models:

1. BLUP-UPG uses ${\mathbf{A}}^{*}$ and is the reference method known to be robust.
2. BLUP-MF uses ${\mathbf{A}}^{\left( \varGamma \right) - 1}$. The main difference is that the latter assumes that MF are random effects and that they are correlated, whereas the former uses UPG that are fixed (unbounded a priori) effects.
3. SSGBLUP-UPG uses ${\mathbf{H}}_{UPG}^{*}$ and is expected to be somewhat biased because it is an approximation.
4. SSGBLUP-EUPG is supposed to be biased also because there is some double-counting. However the bias is not necessarily the same as in SSGBLUP-UPG.
5. SSGBLUP-MF is supposed to be the most accurate method.

All genetic evaluations were performed with heterf90 (not publicly released), which solves the outer model for heterogeneity of variances as in [25], whereas inner iterations used blup90iod2 [35]. To estimate the relationships across MF, we used gammaf90 (not publicly released), which uses the GLS method in [30].

## Results

The estimated value of ${\varvec{\Gamma}}$ is presented below (each row/column corresponds to MF separated by 3 years). We did not explore these values in depth since it was out of the scope of this paper, but, in general, values showed moderate relationships across MF, i.e. most correlations obtained as ${\varvec{\Gamma}}_{{\left( {i,j} \right)}} /\sqrt {{\varvec{\Gamma}}_{{\left( {i,i} \right)}} {\varvec{\Gamma}}_{{\left( {j,j} \right)}} }$ ranged from 0.5 to 0.6. The second and third MF present somewhat extreme values because they have few genotyped descendants. For instance, if the allele frequencies in the base generation were uniformly distributed, the expected value in the diagonal is 2/3 [36]. Matrix ${\varvec{\Gamma}}$ is estimated from estimates of allele frequencies in the base population with standard errors ranging from 0.15 to 0.33, which are the highest values for the second and third MF. These errors seem large but we take the estimate of ${\varvec{\Gamma}}$ as a crude guess, i.e. just as breeding programs start with guessed heritabilities.

$${\varvec{\Gamma}} = \left[ {\begin{array}{*{20}c} {0.53} & {0.24} & {0.37} & {0.38} & {0.39} & {0.39} & {0.41} & {0.41} & {0.41} & {0.42} & {0.44} & {0.43} & {0.40} \\ {} & {0.92} & {0.24} & {0.30} & {0.37} & {0.37} & {0.37} & {0.38} & {0.39} & {0.40} & {0.39} & {0.39} & {0.37} \\ {} & {} & {0.96} & {0.39} & {0.33} & {0.39} & {0.37} & {0.38} & {0.38} & {0.39} & {0.38} & {0.38} & {0.38} \\ {} & {} & {} & {0.72} & {0.37} & {0.34} & {0.37} & {0.38} & {0.37} & {0.38} & {0.37} & {0.39} & {0.37} \\ {} & {} & {} & {} & {0.81} & {0.36} & {0.36} & {0.38} & {0.39} & {0.39} & {0.39} & {0.40} & {0.37} \\ {} & {} & {} & {} & {} & {0.68} & {0.38} & {0.37} & {0.38} & {0.39} & {0.39} & {0.40} & {0.38} \\ {} & {} & {} & {} & {} & {} & {0.69} & {0.39} & {0.38} & {0.38} & {0.40} & {0.41} & {0.38} \\ {} & {} & {} & {} & {} & {} & {} & {0.61} & {0.39} & {0.39} & {0.40} & {0.40} & {0.38} \\ {} & {} & {} & {} & {} & {} & {} & {} & {0.63} & {0.40} & {0.41} & {0.39} & {0.38} \\ {} & {} & {} & {} & {} & {} & {} & {} & {} & {0.59} & {0.42} & {0.41} & {0.39} \\ {} & {} & {} & {} & {} & {} & {} & {} & {} & {} & {0.52} & {0.43} & {0.40} \\ {} & {} & {} & {} & {} & {} & {} & {} & {} & {} & {} & {0.83} & {0.41} \\ {} & {} & {} & {} & {} & {} & {} & {} & {} & {} & {} & {} & {0.59} \\ \end{array} } \right]$$

As for the LR method, Table 1 shows the values of estimators *within* models, i.e. when the model to estimate EBV_p_ and EBV_w_ were the same. In this case, the smallest bias ($\hat{\Delta }$ of 0.23 genetic standard deviations ($\sigma_{g}$) and 0.25 $\sigma_{g}$ for SSGBLUP-MF and BLUP-MF, respectively) was obtained with MF. All models are slightly biased and overestimate the genetic trend (around 0.25 genetic standard deviations, equivalent to 1 year of selection).

**Table 1:** Average $\hat{\Delta }_{p}$ (expressed as $\sigma_{g}$), $\hat{b}_{p}$, $\hat{\rho }_{wp}$, $\hat{\rho }_{pw}^{2}$, $\widehat{acc}_{p}^{2}$ and $\widehat{rel}_{p}$ within models

Model	$\hat{\Delta }_{p}$	$\hat{b}_{p}$	$\hat{\rho }_{wp}$	$\hat{\rho }_{pw}^{2}$	$\widehat{acc}_{p}^{2}$	$\widehat{rel}_{p}$
BLUP-MF	0.25	0.98	0.56	0.32	0.22	0.53
BLUP-UPG	0.48	0.96	0.54	0.31	0.24	0.54
SSGBLUP-MF	0.23	0.97	0.66	0.45	0.32	0.59
SSGBLUP-UPG	0.32	0.94	0.64	0.43	NA	NA
SSGBLUP-EUPG	0.48	0.88	0.61	0.42	NA	NA

Standard errors for all values ≤ 0.01

**Fig. 1:**
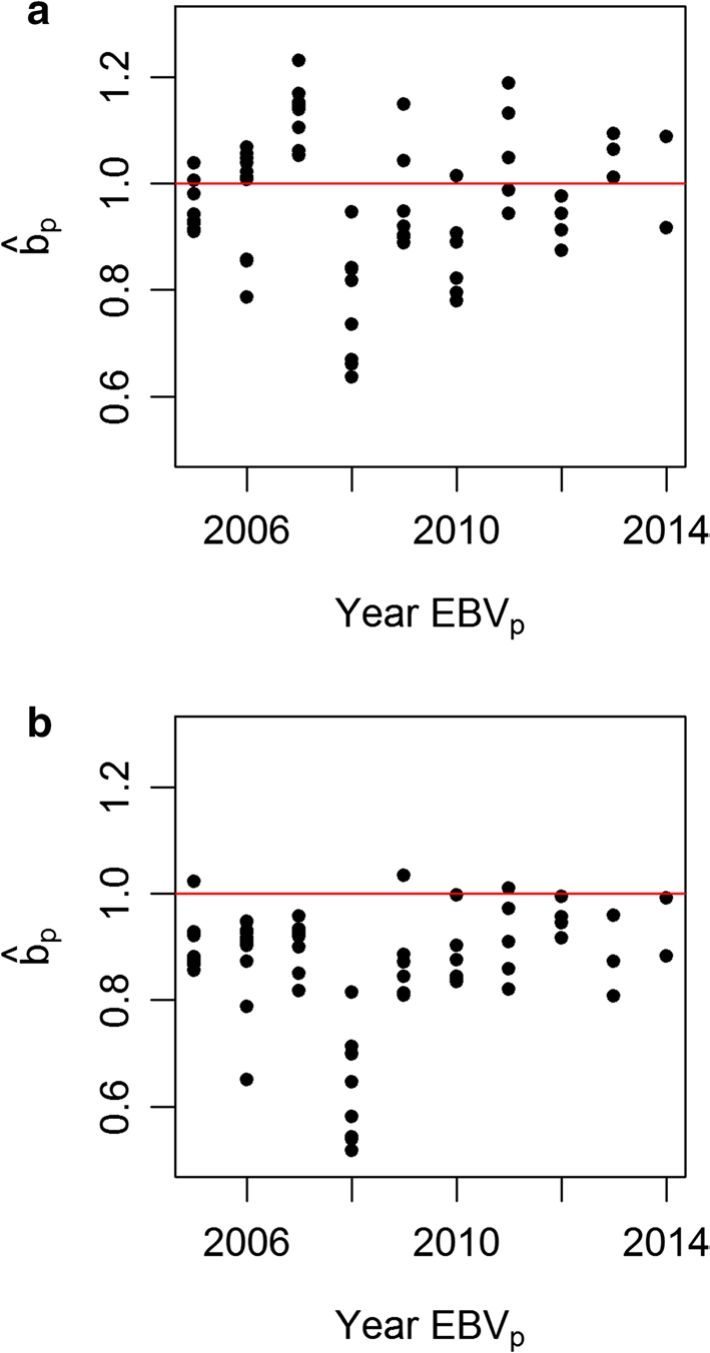
Estimates of $\hat{b}_{p}$ for models BLUP-MF (**a**) and SSGBLUP-EUPG (**b**) by year of EBV_p_ evaluated

For the estimator of dispersion ($\hat{b}_{p}$), for all models, except for SSGBLUP-EUPG, the values were close to 1, meaning absence of over- or under-dispersion of EBV. However, SSGBLUP-EUPG model was biased ($\hat{b}_{p} = 0.88$), which indicates inflation of EBV. This agrees with [18] who found that SSGBLUP-EUPG was biased. In Fig. 1, we present the values of each estimate of $\hat{b}_{p}$ for BLUP-MF (Fig. 1a), which has the average value of $\hat{b}_{p}$ closest to 1, and for SSGBLUP-EUPG (Fig. 1b), which generates the most over-dispersion. The variability of the estimates of EBV_p_ within and across years is similar for both models, but the estimates of dispersion with SSGBLUP-EUPG are systematically the smallest. As Fig. 1 shows, the year of birth 2008 seems to yield biased estimators. This agrees with [14] who found biases for predictions of rams born in this year. Figure 1 also illustrates that there is a large variability of estimates within and across years of the “partial” and “whole” datasets, with the implication that a single time-point is not sufficient to describe the behavior of the genetic evaluation.

Estimator $\hat{\rho }_{wp}$ represents the inverse of the relative gain in accuracy from EBV_p_ to EBV_w_, thus high values of this estimator imply higher accuracy in the “partial” dataset, as expected for SSGBLUP. In agreement, values of this estimator were lower for the BLUP models (roughly 0.55) than for the SSGBLUP models (roughly 0.65). In other words, the EBV of the rams obtained without the records of theirs daughters were more accurate in SSGBLUP than in BLUP, which agrees with [14]. Similar results were found for $\hat{\rho }_{pw}^{2}$, which estimates the ratio between reliabilities in EBV_p_ and EBV_w_.

The direct estimators of accuracy ($\widehat{acc}_{p}^{2}$ and $\widehat{rel}_{p}$), both based on the covariance between EBV_p_ and EBV_w_, presented extremely high values (in some cases, the variance of EBV_w_ was larger than the genetic variance), for SSGBLUP-UPG and SSGBLUP-EUGP, and are therefore not reported. This may be an indirect indicator of the poor fit of UPG to SSGBLUP, whereas BLUP-UPG shows reasonable values that agree with the other estimates of accuracy. For BLUP models, $\widehat{acc}_{p}^{2}$ values were lower than for SSGBLUP-MF (0.24 vs 0.32), which agrees with the information obtained from the other estimates of accuracy. Although these values are apparently small, this is expected because this is a sample of animals that are selected based on parent average [20]. In contrast, the estimation of “unselected” reliabilities, $\widehat{rel}_{p}$, results in values within the usual scale of individual model-based accuracies. Again, the SSGBLUP-MF model estimated higher reliabilities than the BLUP models (0.59 vs 0.54 and 0.53, respectively). The increase in accuracy is fairly consistent across all four estimators of accuracy.

In Table 2, we presented the values of the slope of the regression of EBV_w_ on EBV_p_ ($\hat{b}_{p}$) when EBV_p_ was estimated with one model and EBV_w_ with another model. This gives some sort of measure of the disagreement across models, i.e. we expect models to behave similarly in terms of biases. Cases that estimate in a “partial” dataset with SSGBLUP and in a “whole” dataset with BLUP are not considered, since they seem unnatural in practice; for instance the decision on which animals to genotype may be based on the information of the whole dataset. When we use pedigree-based models to estimate EBV_p_ and EBV_w_, the dispersion is around 1 (0.93 and 1.01), regardless of whether UPG or MF are used.

**Table 2:** Average $\hat{b}_{p}$ when EBV_p_ was estimated with one model and EBV_w_ with other model

${\text{EBV}_{\text{p}}}$	${\text{EBV}_{\text{w}}}$				
	BLUP-MF	BLUP-UPG	SSGBLUP-EUPG	SSGBLUP-MF	SSGBLUP-UPG
**BLUP-MF**	0.98	1.01	1.29	0.98	1.32
**BLUP-UPG**	0.93	0.96	1.23	0.92	1.25
**SSGBLUP-EUPG**			0.88	0.61	0.84
**SSGBLUP-MF**			1.28	0.97	1.31
**SSGBLUP-UPG**			0.92	0.69	0.94

Standard errors for all estimations between 0.01 and 0.02. Diagonal include $\hat{b}_{p}$ when both EBV_p_ and EBV_w_ were estimated with the same model

When EBV_p_ were estimated with the BLUP models and EBV_w_ with SSGBLUP-UPG or SSGBLUP-EUPG (the case when genomic selection is implemented), we observed an important under-dispersion (around 1.25). However, SSGBLUP-MF yielded $\hat{b}_{p}$ values close to 1. Similar results were obtained when we compared EBV of the rams at birth, estimated with the BLUP models as “partial” with those estimated with the SSGBLUP models as “whole” (Table 3). The models SSGBLUP-UPG and SSGBLUP-EUPG show important under-dispersion whereas SSGBLUP-MF results in values of $\hat{b}_{p}$ close to 1. This indicates that if we want to change a pedigree-based genetic evaluation for one that includes genomic information, the use of MF is a better option. Moreover, SSGBLUP-EUPG is biased with itself as shown in Table 1, perhaps due to poor compatibility with the ${\mathbf{G}}$ matrices, because of double-counting, or both.

**Table 3:** Average (standard deviation) of $\hat{b}_{p}$ when EBV_p_^*^ was estimated with BLUP and EBV_w_^*^ was estimated with SSGBLUP

EBV_p_	EBV_w_		
	SSGBLUP-UPG	SSGBLUP-EUPG	SSGBLUP-MF
**BLUP-UPG**	1.27 (0.06)	1.21 (0.07)	0.93 (0.05)
**BLUP-MF**	1.34 (0.06)	1.27 (0.07)	0.98 (0.05)

## Discussion

This study provides a comprehensive analysis of bias, dispersion and accuracies in dairy sheep genetic evaluation with several truncation points of data and several models. Estimates of bias, dispersion and accuracy were obtained with evaluation models that used only pedigree or a combination of pedigree and genomic relationship matrices with different strategies to model missing pedigree and using the LR method. The properties of such types of models have recently been extensively investigated [18, 30, 36–40]. The current study adds further evidence that the metafounder approach should be the preferred one for genomic evaluation across species.

The values of accuracy estimators confirm that the inclusion of genomic information increases the accuracy of the EBV of individuals without daughter records, which is consistent with other studies [41–44].

For $\widehat{acc}_{p}^{2}$, we found extremely high values for models SSGBLUP-UPG and SSGBLUP-EUPG, due to values out of the parametric space. For example, for SSGBLUP-UPG and the comparison 2010–2015, $cov\left( {\hat{u}_{p} ,\hat{u}_{w} } \right) = 235$, $var\left( {\hat{u}_{p} } \right) = 283$ and $var\left( {\hat{u}_{w} } \right) = 580$, when the genetic variance in the base population is 565. This could indicate a difficulty for these models to manage correctly missing pedigree through UPG and the genomic information. Values within the expected range of reliabilities were found for the other models, and the SSGBLUP-MF model reached the highest average value. These results agree with the values of estimators of the ratio of accuracies ($\hat{\rho }_{wp}$ and $\hat{\rho }_{p}^{2}$), since the use of genomic information increases the reliability of EBV estimated without daughter records. We should note that $\widehat{acc}_{p}^{2}$ tries to estimate the square of the correlation between EBV and TBV in the focal individuals, that are selected and with reduced variance, whereas $\widehat{rel}_{p}$ would be the squared correlation if they were unselected. These two estimators have different purposes in practice [20]: the first, populational reliability $\widehat{acc}_{p}^{2}$, describes the possible genetic gain, whereas the second describes stability of EBV. In the current breeding scheme of the Manech Tête Rousse, more candidates are genotyped for selection, so that our estimate $\widehat{acc}_{p}^{2}$ is possibly a lower bound.

Concerning the bias ($\hat{\Delta }_{p}$), the lowest values were observed when MF were used to model the missing pedigree. As for the estimator of dispersion ($\hat{b}_{p}$), we did not observe important over- or under-dispersion, except for SSGBLUP-EUGP. The closest values to 1 of this estimator were obtained when we used BLUP-MF and SSGBLUP-MF. Similar results were obtained in a recent work [18], which indicates that MF could be the best option to manage missing pedigree for SSGBLUP models. In the case of SSGBLUP-EUPG, an important inflation of EBV was observed. A possible cause for this behavior could be that EUPG ignores the covariance between genetic groups (average relationship across MF is 0.38) whereas this relationship is included in ${\mathbf{G}}$. Similar results were reported by [18] using simulated data to compare the same three strategies to model missing parents, and they found that MF generated the smallest bias in evaluations.

In general, when BLUP or SSGBLUP_MF were used, no bias was found, although Legarra et al. [14] found biases in these same breeds using DYD both as pseudo-phenotypes and for validation. However, as we already mentioned, the validation set in [14] was composed of rams born in 2008–2009 with predictions that were also biased according to the LR method, which was due to a problem in collecting elite rams across flocks.

Finally, we consider important to highlight that a single cut-off point to estimate accuracy or bias is highly uncertain, as shown in Fig. 1. Breeding schemes should not rely on a single study based on a single point in time to define models for genetic evaluation.

## Conclusions

The addition of genomic information increases the accuracy of the EBV of young rams in Manech Tête Rousse. In this population, that has missing pedigrees, the use of UPG and “exact UPG” in SSGBLUP produced bias, whereas MF yielded unbiased estimates and, thus we recommend its use. We also recommend assessing biases and accuracies using multiple truncation points, as these statistics are subject to random variation.

## Data Availability

The data set is available under reasonable request.

## Appendix

### R code to obtain estimable functions of statistics in LR method

We used function estimable() from the R package Gmodels v. 2.18.1

#https://www.rdocumentation.org/packages/gmodels/versions/2.18.1/topics/estimable

library(gmodels)

# read input file

input = read.table(“input.txt”)

# 1 represent the intercept, 9 is number of years in “partial” minus one (included in the intercept), 10 is number of years in “whole” minus one (included in the intercept) cm = c(1,rep(1/9,9),rep(1/10,10))

# example with bias

lm_bias = lm(bias ~ as.factor(y_partial) + as.factor(y_whole),data = input)

# estimable function

estimable(lmbias,cm,conf.int = 0.95)
